# Cross-cultural research of coping: Individualization and modernization in Post-Soviet societies

**DOI:** 10.1192/j.eurpsy.2021.1813

**Published:** 2021-08-13

**Authors:** V. Lianguzova, D. Khoroshilov, E. Belinskaya

**Affiliations:** 1 Psychology, Moscow State University, Moscow, Russian Federation; 2 Faculty Of Psychology, Russian State University for the Humanities, Moscow, Russian Federation

**Keywords:** Qualitative methods, Culture, Ethnopsychology, coping

## Abstract

**Introduction:**

The article is devoted to the main methodological problems of qualitative research in the psychology of culture. Methods of ethnographic (field) observation were borrowed by psychology from social and cultural anthropologists in the first half of the twentieth century, and in modern research practices there is a special ethnographic direction that claims to analyze behavior and lifestyle in various subcultures and communities.

**Objectives:**

The purpose of this study was to identify the key cross-cultural difference in coping behavior of respondents from Moscow and Tashkent.

**Methods:**

The methodological basis of the research was the thematic analysis of narratives and free-form interviews. The sample was N = 60 (residents of Russia and Uzbekistan age 17-39).

**Results:**

The results of thematic analysis of narratives and free-form interviews of respondents from Moscow and Tashkent allow us to conclude that the key cross-cultural difference in coping behavior is the degree of its individualization: representatives of Uzbek culture are focused on receiving support and care from significant Others, and not on independent internal work (unlike Russian respondents). At the same time, they are not satisfied with the traditional prescriptions that come from the family environment, which forces them to coping practices that go beyond the boundaries of normative social (often religious) ideas.

**Conclusions:**

This can be interpreted from the point of view of the process of modernization of Uzbek culture, which is gradually becoming individualistic, and the latter circumstance requires the construction of flexible coping strategies in the situation of social and cultural changes.

**Disclosure:**

No significant relationships.

